# The *lin-35/*Rb and RNAi pathways cooperate to regulate a key cell cycle transition in *C. elegans*

**DOI:** 10.1186/1471-213X-7-38

**Published:** 2007-04-27

**Authors:** Jimmy Ouellet, Richard Roy

**Affiliations:** 1 Department of Biology, McGill University, 1205 Dr. Penfield, Montréal, H3A 1B1, Canada

## Abstract

**Background:**

The Retinoblastoma gene product (Rb) has been shown to regulate the transcription of key genes involved in cell growth and proliferation. Consistent with this, mutations in Rb are associated with numerous types of cancer making it a critical tumour suppressor gene. Its function is conferred through a large multiprotein complex that exhibits a dual function in both activation and repression of gene targets. In *C. elegans*, the Rb orthologue *lin-35 *functions redundantly with other transcriptional regulators to appropriately specify both vulval and pharyngeal cell fates.

**Results:**

In *C. elegans *the intestinal cells must alter their cell cycle from the mitotic cell divisions typical of embryogenesis to karyokinesis and then endoreplication, which facilitates growth during larval development. While screening for genes that affect the ability of the intestinal cells to appropriately make this cell cycle transition during post-embryonic development, we isolated mutants that either compromise this switch and remain mononucleate, or cause these cells to undergo multiple rounds of nuclear division. Among these mutants we identified a novel allele of *lin-35/*Rb, while we also found that the components of the synMuv B complex, which are involved in vulval specification, are also required to properly regulate the developmentally-controlled cell cycle transition typical of these intestinal cells during larval development. More importantly, our work uncovered a role for certain members of the pathways involved in RNAi in mediating the efficient transition between these cell cycle programs, suggesting that *lin-35*/Rb cooperates with these RNAi components. Furthermore, our findings suggest that *met-2*, a methyltransferase as well as *hpl-1 *and *hpl-2*, two *C. elegans *homologues of the heterochromatin protein HP1 are also required for this transition.

**Conclusion:**

Our findings are consistent with *lin-35*/Rb, synMuv and RNAi components cooperating, probably through their additive effects on chromatin modification, to appropriately modulate the expression of genes that are required to switch from the karyokinesis cell cycle to endoreplication; a highly specified growth pathway in the intestinal epithelium. The *lin-35/Rb *repressor complex may be required to initiate this process, while components of the RNAi machinery positively reinforce this repression.

## Background

Multicellular organisms must continuously integrate information from the developmental program in order to appropriately coordinate cell divisions with cell fate specification to generate functional tissues and organs. These developmental cues often impinge directly on key positive and/or negative regulators of the cell cycle. Although other levels of regulation exist, in many cell types the critical point resides with the decision to execute S phase. Several key growth-promoting signal transduction pathways have been implicated in this decision, most of which act upon the levels or the activity of cyclin D/CDK4/6 complexes during G1 phase [1]. Perhaps the best characterized among the many important substrates of this kinase is the tumor suppressor gene Rb, which was originally identified as a gene commonly mutated in rare heritable retinoblastomas [2]. Since its initial identification, this gene product has been implicated in many different types of cancer and compromise of its function may be one of the most common events preceding the onset of tumorigenesis [3].

Rb is an important negative regulator of cell growth and proliferation and orthologues have been identified in most metazoans from *C. elegans *to humans and more recently in plants [2,4-6]. These proteins possess a characteristic "pocket domain" that is required for their essential tumor suppressor activities. Initially, Rb was described as a negative regulator of the S-phase specific transcription factor E2F and through its action maintains the cell in G1 phase. The tumor suppressor function associated with Rb may, at least in part, be mediated through this important property. However, our understanding of the role of Rb and the related pocket proteins has expanded and it appears that their functions are not confined to regulating the cell cycle. Rather, the pocket-containing proteins are now known to play important roles other than in cell cycle regulation in several developmental contexts in many different organisms [7-9].

A developmental role of Rb became apparent following the analysis of Rb -/- mice that died early during embryogenesis from various developmental abnormalities which were difficult to account for solely based on cell cycle defects [10-12]. Subsequently, Rb was found to associate with known key transcription factors required for organ specification, thus confirming the role of Rb in regulating genes required for other essential cellular processes [9]. The Rb/E2F/DP1 complex not only plays an important developmental role in mice, but its developmental role is also conserved in invertebrates. In *Drosophila melanogaster*, microarray experiments have revealed that several key developmental regulators appear to be targets of E2F and Rb [13].

In *C. elegans, lin-35 *is the only predicted Rb/pocket protein orthologue and it acts redundantly with additional gene products in several developmental contexts [6]. During embryogenesis, *lin-35*/Rb cooperates with components of the ubiquitin conjugation machinery to specify appropriate pharyngeal morphogenesis [14,15]. Genetic analysis of vulva development has also shown that *lin-35/*Rb is required in the hypodermis rather than in the vulval cells themselves to suppress the ability of the vulval progenitors to respond to sub-threshold *ras *signalling thereby ensuring the correct patterning of the organ [6,16,17]. Because of the redundant pathways that cooperate with Rb to control these transcriptional outcomes in the vulva, the mutants in these pathways have been referred to as synthetic Multivulval or synMuv. At present, three different classes of synMuv genes have been characterized based on their genetic interactions and are referred to as either synMuv A, B or C, all of which act in parallel to appropriately pattern the vulva [18,19]. *lin-35*/Rb was initially characterized as a synMuv B gene, as were the E2F orthologue *efl-1 *and its binding partner *dpl-1 *[6,20]. Not unexpectedly, many of the synMuv B genes are involved in various aspects of transcriptional control.

Biochemical and genetic evidence suggests that Rb does not only sequester E2F transcription factors at the G1 phase of the cell cycle, but it also forms a functional repressor complex with E2F and its DNA-binding partner DP, in addition to several other proteins that together repress the expression of genes at various points during the cell cycle [21]. The notion that Rb, E2F and DP reside together in a complex involved in modulating gene expression was further supported when components of a functional *myb *transcription factor-containing complex were identified in *Drosophila*. Among the associated polypeptides identified in this complex were the *Drosophila *synMuv B orthologues, suggesting that these proteins form a functional complex that represses gene expression in various developmental contexts in different organisms [22,23]. Further evidence in support of a repressive function for the Rb complex was provided from findings that demonstrated that Rb interacts directly with Suv39h, a methyltransferase specific for lysine 9 on histone 3 [24]. This methylated lysine creates a binding site for the heterochromatin protein HP1, which promotes the formation of heterochromatin [25,26]. Therefore, the tumor suppressor function of Rb proteins may equally depend on their ability to repress the expression of key developmental genes in cooperation with E2F and DP, in addition to its well-characterized role in regulating E2F activity for the expression of the repertoire of genes associated with the onset of S-phase.

In *C. elegans*, the *lin-35*/Rb complex was demonstrated to regulate cell division in the intestinal lineage by cooperating with *cki-1/-2*, two cyclin-dependant kinase inhibitors [27,28]. However, it was not clear at what level *lin-35/*Rb was required, particularly since the intestinal cells undergo 3 different invariant cell divisions during their development. Since these transitions are under developmental control, we conducted a forward genetic screen to identify genes involved in determining appropriate transitions between these different cell cycles typical of the intestine during post-embryonic development of *C. elegans*. We identified several mutants, one of which results from a mutation in *lin-35/Rb *that affects the ability of the intestinal cells to initiate endocycles at the appropriate time during development. In addition to the canonical transcriptional regulators that constitute the synMuv B family, we also found that *met-2*, a predicted methyltransferase, and *hpl-1 *as well as *hpl-2*, two heterochromatin protein-like (*hpl*) that bind methylated histone, also enhance the *lin-35 *mutant phenotype in the intestine. Our data are consistent with *lin-35/Rb *and its associated synMuv B partners being involved in the regulation of gene expression associated with this cell cycle transition.

Moreover, further genetic analysis indicated that the Rb pathway interacts genetically with components of the various RNAi pathways, suggesting that these pathways function additively to regulate the expression of key genes that underlie this developmentally-regulated cell cycle transition. Taken altogether our data provide a link between the synMuv B repressor complex, the RNAi pathways, and chromatin modification in regulating this developmentally-controlled cell cycle transition during post-embryonic development.

## Results and Discussion

The intestinal cells of *C. elegans *execute three different types of cell cycle to give rise to a functional gut in the growing larva. During embryogenesis, the E blastomere undergoes 5 successive rounds of mitotic division which are controlled by both positive and negative cell cycle regulators that are contributed maternally [29,30]. Following embryogenesis, the intestine is highly differentiated and functional to allow the animal to ingest nutrients and therefore initiate larval development. However the larva, as well as the intestinal cells, must continue to grow as the animal progresses through post-embryonic development. This represents a fundamental problem for the animal, as terminal differentiation and cell proliferation are generally mutually exclusive cellular states. To circumvent this, the cells execute a single nuclear division during the first larval stage and then execute "endocycles", where they fully duplicate their genome without undergoing cytokinesis in order to coordinate their growth with the feeding larva, while still maintaining a fully differentiated and functional gut.

**Figure 1:**
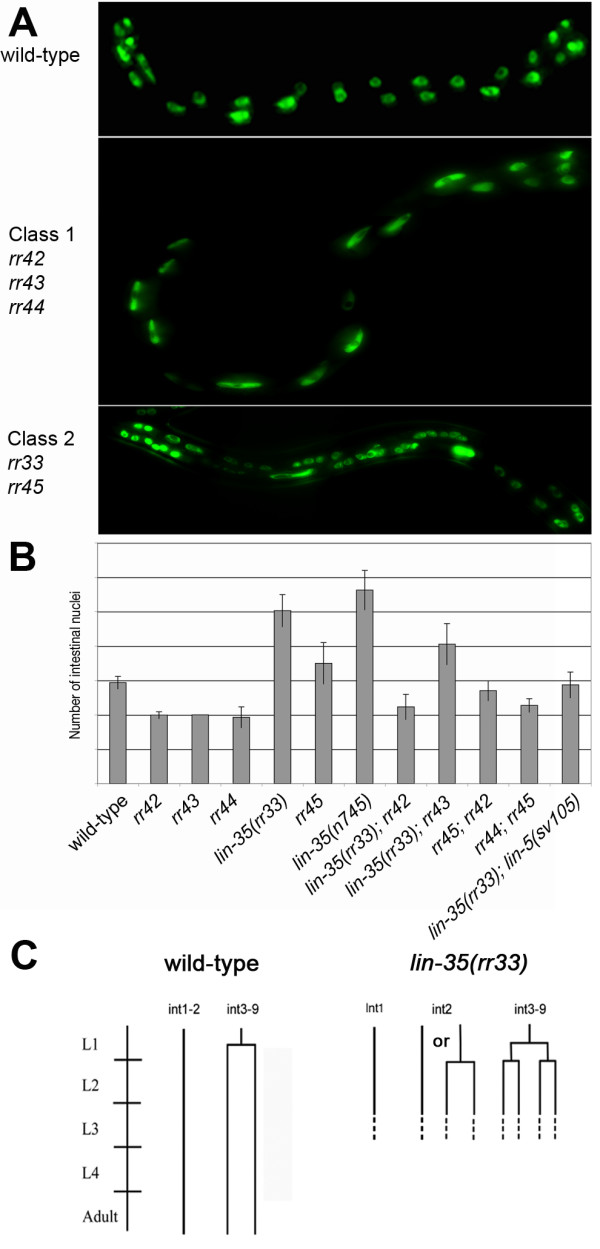
Two classes of mutants with altered numbers of intestinal nuclei. (A) Shows a wild-type (upper panel), a class 1 mutant with fewer than the wild-type number of intestinal nuclei (*rr44*) (middle) and class 2 mutant with extra intestinal nuclei (*lin-35(rr33)*) (lower panel). All animals were at the L4 larval stage and express the intestinal-specific *elt-2::*GFP. Anterior is left in all pictures. (B) Genetic interaction between the class 1 and class 2 mutants. All mutants with less intestinal nuclei (class 1) are epistatic to the mutants with supernumerary nuclear divisions (class 2). The double mutant *lin-35(rr33); rr45 *is synthetic lethal, therefore it is not represented on this graph. (C) Lineage analysis of the intestinal cells in *lin-35(rr33) *mutants. The diagram is representative of observations collected from 8 independent larvae from the initiation of post-embryonic development up to the end of the L2 stage. Horizontal lines represent intestinal nuclear divisions, while the vertical lines represent time.

Using an intestinal-specific GFP marker (*elt-2::GFP*) to isolate mutants with an aberrant number of intestinal nuclei we have previously shown that appropriate activity of *cki-1*, the *C. elegans *p21/27-like cyclin dependent kinase inhibitor and the positive S-phase regulator *cdc-25.1 *are critical to control cell divisions in the intestinal cells during embryogenesis, without any effect on the post-embryonic nuclear divisions or endocycles [29,30]. In a similar screen performed to identify genes involved in the post-embryonic cell cycle transitions we isolated five mutants that either possess fewer intestinal nuclei than wild-type (*rr42, rr43 *and *rr44*, class 1 mutants), or more than the wild-type complement of intestinal nuclei (*rr33 *and *rr45*, class 2 mutants) (Figure 1A). Initial characterization of these mutants indicated that they all exhibit a maternal effect, with the exception of *rr45*. The mutants that give rise to extra intestinal nuclei, *rr33 *and *rr45*, are temperature sensitive and produce only a few eggs before they become sterile when transferred to restrictive temperature (25°C). Some of these embryos hatch, but these larvae arrest post-embryonic development before the L2 stage. Both class 1 and 2 mutants hatch with the wild-type number of intestinal cells (20 cells) (Table 1), indicating that these mutations do not affect embryonic divisions, unlike *cki-1 *or *cdc25.1(gf)*, but they specifically affect the post-embryonic nuclear divisions.

**Table 1:** Characterization of class1 and 2 mutants with altered numbers of intestinal nuclei.

**Class 1**				
Genotype	Number of nuclei		Genetic	Map location
			
	L1	Adult		

*rr42*^1^	20 ± 1	21 ± 1	Recessive, maternal	LG V, btwn 2.52–2.66
*rr43*^1^	20	21 ± 2	Recessive, maternal	LG V, btwn 2.52–2.66
*rr44*	19 ± 1	21 ± 2	Recessive, maternal	LG I, btwn 2.2–2.6

**Class 2**				

*rr33*	20	50 ± 5	Recessive, maternal	LG I, 0.36 MU
*rr45*	20	35 ± 6	Recessive, zygotic	LG IV, 3.46 MU

^1 ^Complementation tests indicates that *rr42 *and *rr43 *are allelic.

The presence of two classes of mutants prompted us to examine the genetic interactions between these different alleles. First, we found that *rr33 *and *rr45 *double mutants are synthetically lethal, suggesting that the genes affected in these two mutants are part of the same essential cellular process, or that they affect two parallel but inter-dependent pathways [31]. We also found that class 1 mutations (less intestinal nuclei than wild-type) suppress both class 2 mutations (more intestinal nuclei-*rr33 *and *rr45*), albeit to different degrees suggesting that the genes encoded by class 1 mutations may be epistatic to those of class 2 (Figure 1B). Although at present we have not uncovered the molecular identity of these mutated genes, the phenotype of class 1 mutants strongly resembles that of gene products involved in the LIN-5 complex [32]. The LIN-5 complex is required during the first cell division to generate force at the mitotic spindle. However, the intestinal cells in hypomorphic alleles of *lin-5 *mutant are not able to perform the nuclear division at the L1 stage and remain mononucleate. Consistent with this, we found that *lin-5 *is also epistatic to *rr33*, much like our class 1 mutants (Figure 1B). At present, our mapping data indicate that these mutations do not correspond to any of the known *lin-5 *complex components (Table 1). Therefore our class 1 mutants may be implicated in a previously undescribed *lin-5*/*GPR*-related pathway(s) for generating force required for the nuclear divisions that occur in the intestine during the first larval stage (L1).

### *rr33 *is a mutation in the Retinoblastoma (Rb) gene orthologue *lin-35*

**Figure 2:**
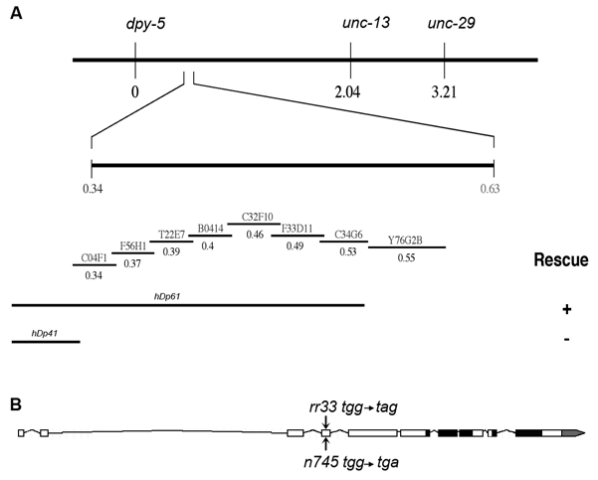
Mapping and cloning of the *rr33 *mutant. (A) SNP-SNIP mapping as well as three-factor mapping approaches were used to map *rr33 *to the center of linkage group I, close to *dpy-5*. This region contains several well-defined free-duplications of which *hDp61 *rescued the *rr33 *mutant (+) whereas *hDp41 *did not (-). C32F10.2(RNAi) phenocopies the *rr33 *mutant phenotype. (B) A schematic representation of the C32F10.2 *lin-35*/Rb gene with black boxes representing the predicted pocket binding domains and the gray box indicating the 3' UTR. The location and the precise nucleotide changes in *rr33 *and *n745 *allele are indicated by arrows.

The class 2 mutants *rr33 *and *rr45 *have similar phenotypes that include supernumerary intestinal nuclei that arise postembryonically. We mapped the *rr33 *mutation to a small interval on chromosome I (Figure 2A), while *rr45 *maps to chromosome IV. Using the feeding RNAi library we tested all the predicted genes in the region to identify candidates, which upon loss-of-function, would phenocopy the *rr33 *mutant phenotype in wild-type animals [33]. Only C32F10.2(RNAi), which corresponds to the *C. elegans *Rb orthologue *lin-35*, fully phenocopied the *rr33 *mutant phenotype when fed to otherwise wild-type animals. LIN-35 is the only pocket protein predicted within the *C. elegans *genome and it was initially identified due to its role in vulva development as a member of the synMuv B class of genes [6]. Mutations in synMuv B genes cause a multivulval (Muv) phenotype only when combined with a class A or C synMuv mutation [18,19].

We sequenced *lin-35*/Rb amplified from the *rr33 *mutant and found a GC to AT transition at nucleotide 442, which changes the codon from TGG to TAG, thereby introducing a premature amber stop codon (Figure 2B). To confirm that *rr33 *was indeed an allele of *lin-35*, we tested it in combination with *lin-8(n111) *a known class A synMuv mutant [34]. Although both mutant backgrounds on their own have no visible vulva defects, all of the *rr33*; *lin-8(n111) *double mutants show the synMuv phenotype (data not shown). Furthermore, the *rr33 *phenotype in the intestine and the lethality at 25°C are both fully rescued using a functional *lin-35::GFP *translational fusion protein (see below and data not shown) [17]. Therefore, based on RNAi data, sequence analysis of the *rr33 *mutation, transformation rescue, as well as its ability to produce a synMuv phenotype, we conclude that *rr33 *is a novel allele of *lin-35 *and will be herein referred to as *lin-35(rr33)*.

Previous findings showed that the reference allele *lin-35(n745) *had no intestinal nuclear division phenotype alone, but acted synergistically with *cki-1/cki-2 *to give rise to extra intestinal cells/nuclei [27,28]. However, this does not seem to be the case as both the reference allele *lin-35(n745) *and this novel allele *lin-35(rr33) *increase the number of intestinal nuclei quite substantially above the wild-type complement in the absence of any other background mutation (Figure 1B) [35]. Furthermore, this phenotype is not likely the cause of second site mutations as similar increases in the number of intestinal nuclei have been observed when both of these mutations are crossed into different genetic backgrounds ([35], and data not shown).

Although it was originally reported that *lin-35 *single mutants exhibit few visible phenotypes other than a reduction in fertility, we find that *lin-35 *is an essential gene. We found that both *lin-35(rr33) *and the previously described reference allele *lin-35(n745) *arrest post-embryonic development at 25°C before they reach adulthood when upshifted at the L1 stage (data not shown), while upshifting at the L4 stage results in adults with a low brood size (average of 66.1 ± 20.8 progeny compared to 169 ± 27 in wild-type, while 38.7% ± 16.9 of these embryo arrest embryonically in *lin-35(rr33) *mutant, n = 10 animals). The few embryos that hatch at 25°C arrest post-embryonically before they reach the L2 stage.

The phenotypic differences observed for *lin-35 *mutants reared at 20°C vs 25°C prompted us to question if *lin-35(rr33) *is indeed a null allele, despite the stop codon that it introduces early in the coding sequence. However, classical tests could not be performed because no deficiencies are currently available that uncover the *lin-35 *locus. The presence of a stop codon motivated us to look at the abundance of the mRNA, as aberrant transcripts are usually degraded by nonsense mediated decay [36]. Using semi-quantitative RT-PCR, we were able to detect *lin-35 *mRNA in *lin-35(rr33) *mutants at levels comparable to wild-type (data not shown). This result suggests that even if the *rr33 *mutation introduces a stop codon, the mRNA is not degraded through the nonsense mediated decay pathway. Also, even if the reference allele of *lin-35, n745*, also introduces a stop codon at the same position (opal rather than the amber stop found in *rr33*), the intestinal phenotype is more pronounced in *lin-35(n745) *compared to *lin-35(rr33) *(56.3 ± 4.7, n = 21 intestinal nuclei compared to 50.7 ± 4.7, n = 38 respectively). This difference between these two alleles is likely due to the varying capacity of readthrough associated with the two different stop codons. In support of this, we can recapitulate the embryonic lethality typical of both alleles when maintained at 25°C by injecting *lin-35 *dsRNA in a *lin-35(rr33) *mutant reared at permissive temperature (40.5 ± 21.1 % embryonic lethality compared to 2.2% embryonic lethality in uninjected *lin-35(rr33) *mutant, n = 10). Our data therefore suggest that *lin-35 *is an essential gene and neither of these alleles act as null mutations at 20°C, presumably due to readthrough.

It was recently shown that *lin-35*/Rb and probably all the other components of the synMuv B complex are required in the hypodermal cells rather than in the vulva cells to prevent inappropriate ras activation [16,17]. Because of this cell non-autonomous effect of *lin-35/*Rb in the vulva cells, we wanted to test if *lin-35 *is required autonomously in the intestinal cells to regulate this cell cycle transition. To address this we expressed a rescuing *lin-35 *cDNA specifically in the intestinal cells using the *elt-2 *promoter in a *lin-35(rr33) *mutant background. We observed that animals that carry the transgene have 32.6 ± 2.8 intestinal nuclei compared to 29.3 ± 1.8 for wild-type. Therefore unlike its role during vulval development, *lin-35 *activity is required autonomously within the intestinal cells to control the intestinal cell cycles characteristic of the L1 stage.

### *lin-35 *is required for the timely initiation of endocycles

The intestine of a wild-type adult *C. elegans *hermaphrodite is composed of 32 nuclei divided among 20 cells due to a single round of nuclear division that occurs in the first larval stage (L1). In contrast, the *lin-35(rr33) *mutant adult intestine possesses ~50 intestinal nuclei. This defect does not occur embryonically since mutant larvae hatch with the normal complement of intestinal cells/nuclei (Table 1). Therefore we performed lineage analysis on *lin-35(rr33) *mutants to determine when these extra intestinal nuclei arise during post-embryonic development. We found that in *lin-35(rr33) *mutants the L1-specific nuclear divisions that occur in intestinal rings 3–9 (Int3-9) are essentially identical to wild-type and the intestinal cells become binucleate (Figure 1C). However, *lin-35(rr33) *mutants undergo a supernumerary round of nuclear division in the posterior intestinal cells in the early L2 stage, just after the normal onset of the endocycle program. However, not all the intestinal cells execute this division (on average, 48.9% of the intestinal cells in each animal are abnormal, n = 20 animals) and there is no bias for any single cell to divide. Moreover, whereas the cells of the Int2 ring never undergo nuclear division in wild-type, these cells will often execute a nuclear division at the L2 stage to become binucleate in the *lin-35(rr33) *mutant background, wherein 22.9% of the *lin-35(rr33) *had at least one divided Int2 cell (n = 20). We interpret these observations to indicate that affected intestinal cells are unable to make the timely transition to endocycles at the L2 molt due to sub-threshold levels of *lin-35*/Rb, thus compromising the appropriate downregulation of specific gene activities. As such, the affected cells undergo an extra round of nuclear division to eventually possess supernumerary intestinal nuclei.

Previously, *lin-35 *was found to cooperate with *cki-1/2 *to regulate intestinal nuclei numbers in the adult [27,28]. However, the presence of extra intestinal nuclei at the adult can be due to both supernumerary mitotic divisions during embryogenesis or nuclear divisions during post-embryonic development. To confirm that indeed *cki-1 *and *lin-35 *are acting in two distinct developmental processes, we inactivated *cki-1 *by RNAi in wild-type and *lin-35(rr33) *mutant backgrounds and scored the number of intestinal nuclei at both the L1 and adult stage. As previously reported, *cki-1(*RNAi) worms hatch with an average of 30 intestinal cells, comparable to the number of intestinal cells in *lin-35(rr33)*; *cki-1(RNAi) *(table 2) [30]. This confirms our lineage analysis indicating that *lin-35 *does not play a role during the embryonic mitotic divisions and does not act in a linear or a parallel pathway with *cki-1 *to control mitotic cell divisions in the intestine during embryogenesis. Therefore, although the loss of both *cki-1 *and *lin-35 *results in considerably more intestinal nuclei in the affected adult animals, this is due to their independent genetic functions in two clearly distinguishable cell cycle processes that occur at distinct times in development [27,28] (Table 2).

**Table 2:** *cki-1 *and *lin-35 *control progression through two different intestinal cell cycle programs during *C. elegans *development.

**Genotype**	**Number of intestinal nuclei ± S.D. (N)**	
	**L1 stage**	**Adult**

**Wild type**	20 (25)	29.4 ± 1.8 (25)
***lin-35(rr33)***	20 (63)	50.3 ± 4.7 (39)
***cki-1(RNAi)***	30.7 ± 2.8 (63)	48.8 ± 5.8 (65)
***lin-35(rr33); cki-1(RNAi)***	31.2 ± 2.4 (43)	92.6 ± 13.65 (45)

### Components of the Rb repressor complex cause abnormalities in the intestinal nuclear divisions

Current models for Rb family function have classed these proteins as negative regulators of the G1/S phase transition of the cell cycle through their ability to sequester transcription factors important for DNA replication such as E2F [37-40]. This interaction is reversed by Rb phosphorylation mediated by cyclin D-CDK4/6 and/or cyclin E/CDK2 activity [41,42]. However, it has been shown that Rb and E2F/DP can form a complex that associates with known transcriptional repressor components such as histone deacetylases to negatively regulate gene expression [23,43,44]. The recently identified components of this Rb-containing repressor complex in *Drosophila *have homologues in human and *C. elegans *and most of these gene products correspond to members of the *C. elegans *synMuv B family [22,23].

**Figure 3:**
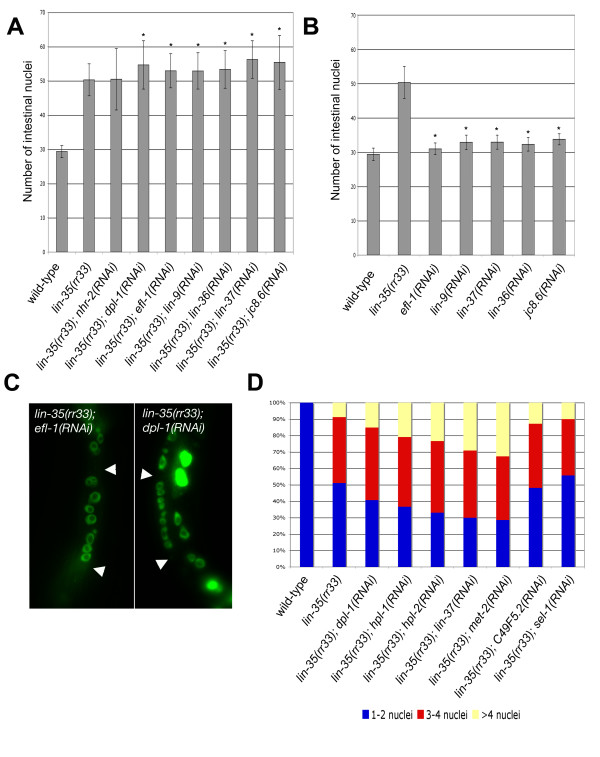
Inactivation of components in the synMuv B gene class enhances the *lin-35(rr33) *intestinal defect. (A) *lin-35(rr33) *animals were fed with bacterial clones that corresponded to the various components of the synMuv B complex and the number of intestinal nuclei were scored in L4 larvae 48 hrs later. The *nhr-2 *gene encodes a nuclear hormone receptor that has not been implicated in the synMuv B complex and was used as a negative control. (B) Feeding RNAi of some of the synMuv B genes was performed on wild-type animals and they also cause an slight increase in intestinal nuclei number, although to a lesser extent than *lin-35(rr33) *alone. The asterisk denotes a Student t-Test value of < 0.05 compared to *lin-35(rr33) *in A and wild-type in B. (C) A representative example of the multinucleate intestinal cells found in double mutant *lin-35(rr33); dpl-1(RNAi) *and *lin-35(rr33); efl-1(RNAi) *animals. The arrowheads indicate the intestinal cell boundaries. (D) The number of multinucleate cells was monitored in various genetic backgrounds. In wild type, all the intestinal cells of the Int3-9 rings have either 1 or 2 intestinal nuclei.

Because of this duality of Rb function, *lin-35 *might be required to prevent the E2F/DP complex from activating S phase specific genes and therefore block nuclear division in the intestinal cells. Alternatively, the LIN-35/E2F/DP repressor complex could act as it does in the vulva cells where it is required to block gene expression [6,20], and in the intestine it would repress the expression of key genes involved in the transition to endocycles. To test these possibilities we performed *efl-1*(RNAi) and *dpl-1*(RNAi) to compromise the E2f and DP orthologues in *C. elegans*, both of which are components of the synMuv B family [20]. Inactivation of both genes through RNAi enhances the intestinal division phenotype of *lin-35 *(Figure 3A and 3C). Because limiting levels of LIN-35 are probably still present in the *lin-35(rr33) *mutant, our results suggest that the activity of this complex is even further reduced when either *efl-1 *or *dpl-1 *are compromised. This would explain how E2F/DP act additively with *lin-35 *to appropriately block nuclear divisions in the intestinal cells at the time they begin to endoreplicate.

To test the importance of the synMuv B complex in regulating this cell cycle transition we performed RNAi against other components of the synMuv B repressor complex and found that all of the synMuv B genes tested enhanced the *lin-35(rr33) *intestinal nuclei phenotype in a manner similar to *efl-1 *and *dpl-1 *(Figure 3A). In *lin-35(rr33); *synMuv B(RNAi) animals many of the affected cells underwent several rounds of intestinal nuclear division to give rise to multinucleate cells that contained more than 4 extra nuclei (Figure 3C and 3D). Control RNAi using genes that are not involved in the synMuv B pathway did not induce any change in the number of intestinal nuclei above the levels observed in *lin-35(rr33) *mutants alone (*nhr-2 *and *sel-1 *in Figure 3A and 3D respectively). We conclude that the synMuv B genes cooperate with *lin-35 *to control the timing of the onset of endocycles, and in their absence the intestinal nuclei undergo repeated rounds of division to eventually give rise to multinucleate intestinal cells.

Not only do the synMuv B components act cooperatively with *lin-35 *to regulate this cell cycle transition, but the reduction of function of these genes alone in a wild-type background causes abnormalities typical of the *lin-35 *nuclear division phenotype, albeit their penetrance and severity is attenuated (Figure 3B). Based on our data and current models of how the Rb regulates gene expression, LIN-35 may act as the core component of a repressor complex that nucleates components of the synMuv B family. The synMuv B components of this Rb repressor complex are important for full activity, but they are individually dispensable. Therefore, the LIN-35/Rb repressor complex can still silence gene expression even in their absence, although not as efficiently as the wild-type complex.

### *lin-35/Rb *is required for the repression of cyclin E

**Figure 4:**
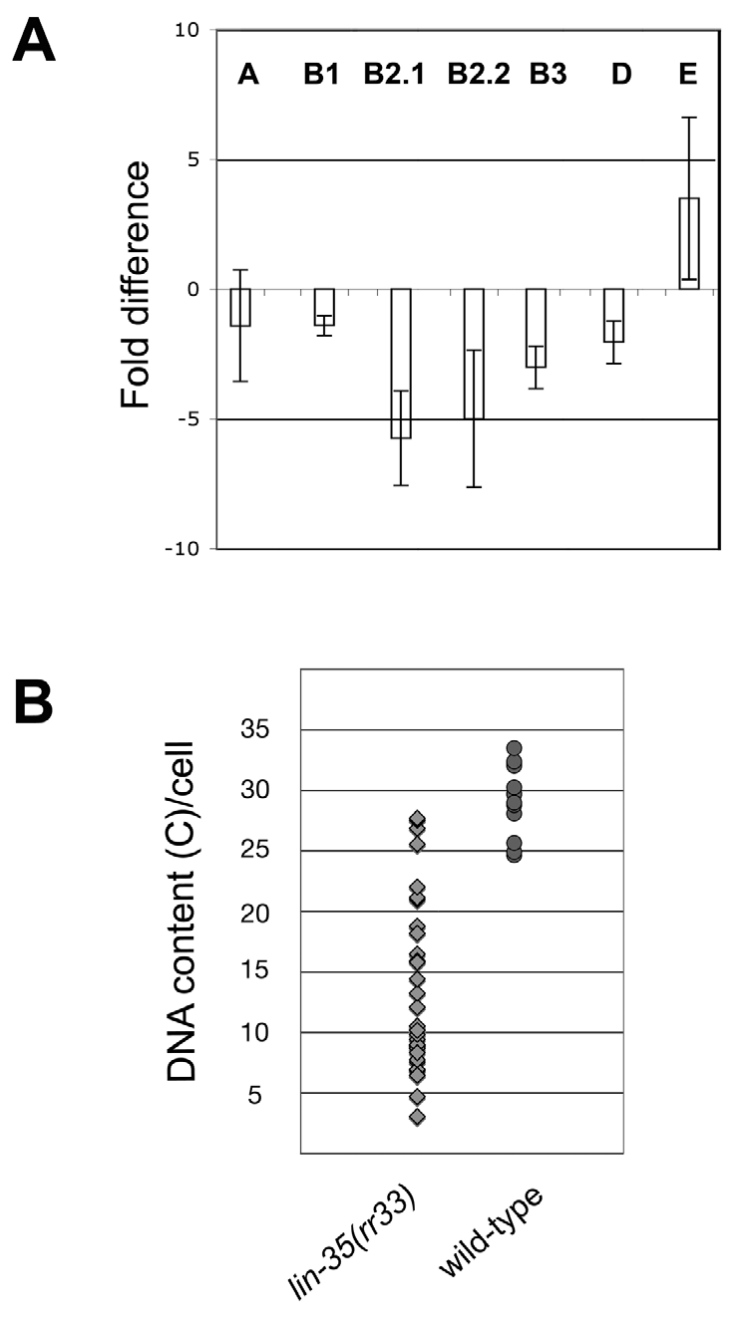
*lin-*35 mutants affect cyclin expression and intestinal nuclear ploidy. (A) qRT-PCR was performed on wild-type and *lin-35(rr33) *L1 larvae and the levels of cyclin expression were determined compared to wild-type. The bars represent 4 independent qRT-PCR reactions from RNA isolated from wild type and *lin-35(rr33) *animals. A positive value indicates an increase in gene expression compared to wild-type, while a negative value indicates a relative decrease in gene expression. The standard deviation is derived from the 4 independent trials. (B) DNA quantification was performed on the intestinal nuclei of both mutant and wild-type young adult hermaphrodites stained with propidium iodide. Each diamond represents a *lin-35(r33) *mutant intestinal nucleus and each square represents an individual wild type intestinal nucleus.

Previously, *lin-35(n745) *was shown to suppress the cell cycle defects associated with mutations in *cyd-1 *and *cdk-4/6 *[27]. Because the *lin-35 *complex is required for transcriptional repression of specific genes, we wanted to determine if the observed effects were a consequence of an increase of cyclin E or cyclin A levels (*cye-1 *and *cya-1 *respectively in *C. elegans*). These two cyclins are known to act downstream of cyclin D and the upregulation of these genes in the *lin-35 *mutant could potentially compensate for the loss of cyclin D. Consistent with this hierarchy, we found that the extra nuclear divisions of the *lin-35(rr33) *mutant are suppressed following *cye-1*(RNAi) (data not shown). In order to test this hypothesis, we performed quantitative (q)RT-PCR to determine if any differences in the levels of A, B, D and E type cyclins were detectable in wild-type *vs lin-35(rr33) *mutants. We observed a significant decrease in both cyclin B2 variants in the *lin-35(rr33) *mutant background compared to wild-type (Figure 4A). We also found that cyclin E showed a 2-fold upregulation in *lin-35(rr33) *mutants (Figure 4A). Cyclin E levels must be tightly regulated to permit only one round of origin firing while providing a cyclic burst of CDK2 activity coincident with the G1/S phase transition [45]. It is therefore quite possible that coupling of the upregulation of *cye-1 *with the observed decrease in B2 cyclins may explain the basis of the supernumerary nuclear divisions and the aberrant ploidy observed in *lin-35(rr33) *intestinal cells (see below) [46].

Recent findings indicate that the Rb complex regulates both cell proliferation as well as endoreplication in mouse trophoblasts and in developing leaves, meristems, and pericycle cells in plants [47-49]. We therefore quantitated the DNA content of the intestinal cells in *lin-35(rr33) *animals to determine if the supernumerary nuclei typical of *lin-35(rr33) *were also associated with an extra full round of DNA replication. If this is the case, the mutant intestinal nuclei should all have a quantized DNA content. This is not the case as we found that the ploidy of the mutant intestinal nuclei in *lin-35(rr33) *ranges from 4C to 26C compared to 32C in wild-type. This result indicates that by disrupting *lin-35 *function, correct genome duplication is compromised and the ploidy of the resulting daughter nuclei is aberrant (Figure 4B). Therefore, *lin-35*/Rb may play a conserved function in timing and/or ensuring the integrity of endoreplication [50].

### Components of RNAi-related pathways cooperate with *lin-35(rr33)*

**Figure 5:**
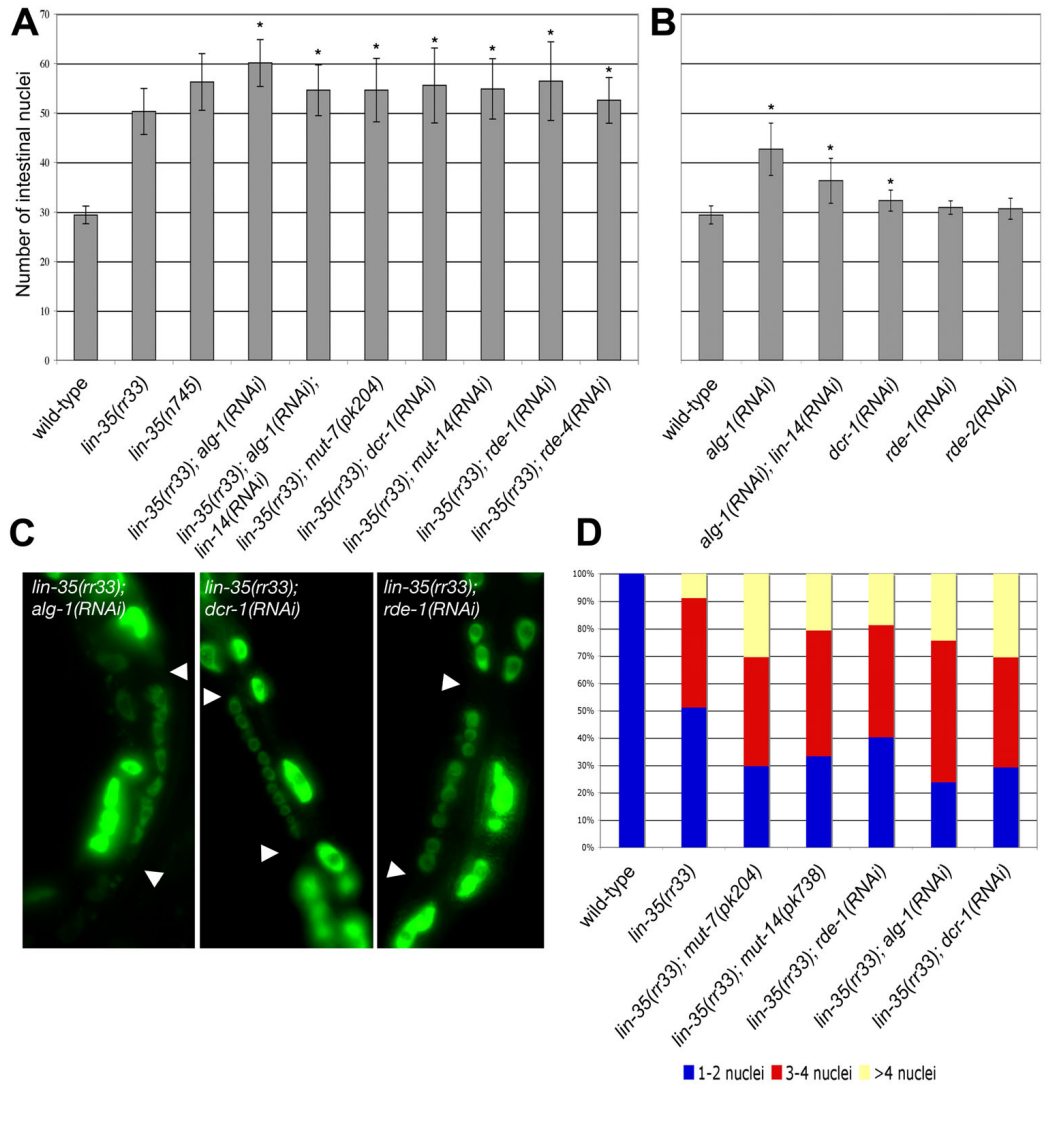
Components of the RNAi pathways cooperate with *lin-35*/Rb to regulate intestinal nuclear divisions. (A) *lin-35(rr33) *L4 animals were fed with bacterial clones that corresponded to the various components of the RNAi pathways and the number of intestinal nuclei was scored in L4 larvae of the next generation. (B) RNAi of some PTGS components was also performed on wild-type worms. The asterisk denotes a Student t-Test value of < 0.05 compared to *lin-35(rr33) *in A and wild-type in B. (C) Examples of the multinucleate intestinal cells typically generated in the double mutants between *lin-35(rr33) *and various components of the RNAi pathways. Some cells can exceed 12 nuclei/per cell. The arrowheads indicate the intestinal cell boundaries. (D) The number of multinucleate cells was monitored in various genetic backgrounds. In wild type, all the intestinal cells of the Int3-9 rings have either 1 or 2 intestinal nuclei.

The reiteration of the intestinal division pattern typical of the L1 stage at the L2 stage in *lin-35(rr33) *mutants is reminiscent of cell cycle abnormalities associated with heterochronic mutants, which are defined by the elimination of entire stage-specific divisions (precocious), or the reiteration of events or cell divisions during larval development (retarded) [51-55]. Control of many of the somatic cell divisions during the L1 stage is dependent on *lin-14 *function and the proper transition to the second larval stage requires the downregulation of *lin-14 *translation through the action of a 22nt miRNA *lin-4 *[56-59]. Mutations in *lin-4 *cause the reiteration of L1-specific cell division program due to the inability to efficiently block *lin-14 *translation. To determine if the *lin-35(rr33) *mutant phenotype could be somehow attributed to a heterochronic defect, we performed RNAi on *alg-1/alg-2 *(the high similarity between *alg-1 *and *alg-2 *at the nucleotide level is likely to target both mRNAs [60]) and *dcr-1 *genes, both of which were shown to be required for *lin-4 *and *let-7 *miRNA processing in *C. elegans *[60]. In wild-type animals *alg-1(RNAi) *or *dcr-1(RNAi) *causes an significant increase in intestinal nuclei, wherein *alg-1(RNAi) *has a much more pronounced effect than *dcr-1(RNAi) *(Figure 5B). However, the incomplete phenotype of *dcr-1*(RNAi) might be due to its essential role in the processing of the dsRNA into smaller 21 nucleotide siRNA, or alternatively, due to the stability of the DCR-1 protein throughout development [61].

Similar to the synMuv B genes, both *alg-1 *and *dcr-1 *(RNAi) enhanced the intestinal phenotype of *lin-35(rr33) *mutants. We found that the intestinal cells in *lin-35(rr33); alg-1(RNAi) *and *lin-35(rr33); dcr-1(RNAi) *double mutants go through several more rounds of nuclear division than *lin-35 *mutants alone to become multinucleate (Figure 5C and 5D). Some adult animals were observed with up to 70 intestinal nuclei compared to 30 in wild type. Because performing RNAi on components required for the efficient execution of this pathway is not always optimal, we tested the effect of an *alg-1 *null allele on the intestinal cells. Surprisingly, the double mutant *lin-35(rr33); alg-1(gk214) *is synthetic lethal, reinforcing the idea that these two pathways interact genetically. It also suggests that despite the very high degree of sequence similarity between *alg-1 *and *alg-2*, *alg-2 *cannot compensate for the loss of *alg-1 *in a *lin-35(rr33) *sensitized background.

To determine whether the observed increase in the number of intestinal nuclei could be due to the decrease in *lin-4 *processing thereby causing the intestinal cells to reiterate the L1 stage division pattern, we performed double RNAi against *alg-1 *and *lin-14*, the known target of *lin-4*. *lin-14(RNAi) *partially suppresses the extra nuclear division caused by *alg-1(RNAi) *in both wild-type and *lin-35(rr33)*, but not to the levels obtained with *lin-35(rr33) *alone (Figure 5A and 5B). Although we cannot rule out the possibility that the *lin-14*(RNAi) does not completely eliminate *lin-14 *mRNA and protein, we suggest that *lin-14 *downregulation by *lin-4 *contributes to the timely transition from karyokinesis to endoreduplication that occurs at the end of the L1 stage.

In addition to its role in processing small RNAs in the heterochronic pathway, *dcr-1 *has also been shown to be a key enzyme involved in RNAi pathways by processing dsRNA into smaller, 21–22 nucleotide small interfering RNA fragments (siRNA) [60]. Our findings that *dcr-1(RNAi) *acted additively with *lin-35 *to enhance the nuclear division phenotype observed in the intestine motivated us to question whether other genes of the RNAi pathway might also genetically interact with *lin-35(rr33) *to affect this cell cycle transition. To test this possibility, we crossed *lin-35(rr33) *into a *mut-7(pk204) *background, one of the known downstream components of the RNAi pathway, and we scored the number of intestinal nuclei [62]. As shown in Figure 5A, *mut-7(pk204) *enhanced the *lin-35(rr33) *extra nuclear division phenotype significantly compared to *lin-35(rr33) *alone (p < 0.005). Since both *dcr-1*(RNAi) and *mut-7(pk204) *enhance the number of extra intestinal nuclear divisions that occur in a *lin-35 *background, we performed RNAi against other known RNAi components to determine if they also caused a similar increase in the number of intestinal nuclei in combination with *lin-35(rr33)*. We found that RNAi against *mut-14, rde-1 *and *rde-4 *also significantly enhanced the number of intestinal nuclei of the *lin-35(rr33) *mutant, while other components such as *rde-2 *did not show any effect (Figure 4A and data not shown). We also scored the number of intestinal nuclei per cell and we observed that the frequency of multinucleate intestinal cells increased at the expense of wild type cells (figure 5D). More importantly, since none of this last group of RNAi components have been shown to be required for miRNA processing or function, we conclude that the defects observed in their absence in the *lin-35 *mutant cannot be exclusively attributed to a heterochronic defect. Although the *lin-35 *and miRNA pathways may be additive, they affect the developmental switch to the growth program in the intestinal cells by independent mechanisms.

Increasing evidence suggests that the RNAi machinery plays an important role in establishing key chromatin modifications to allow stable repression of gene expression [63-67]. One of the best characterized modifications is methylation of lysine 9 on histone 3. This is catalyzed predominantly by the methyltransferase Suv39h, which has been implicated in the initiation of heterochromatin formation in several cell types and in different organisms [68]. Once methylated, heterochromatin proteins (HP) such as HP1 recognize the modification and bind to the methylated histone to induce conformational changes in the chromatin typical of heterochromatin. *C. elegans *has two HP1-like proteins (HPL-1 and HPL-2) and *hpl-2*, but not *hpl-1*, was shown to be a synMuv B gene [69]. This suggests that the synMuv B repressor complex may induce chromatin changes by binding target gene promoters, which are subsequently methylated. This step could occur either through direct association of a methyltransferase with the repressor complex, or in a subsequent step in response to its binding. Recently, *met-2*, a predicted meL9H3 methyltransferase, was found to behave as a synMuv B class gene, reinforcing the idea that the synMuv B genes induce transcriptional repression through chromatin modification [70]. Consistent with this, we found that when performed in a *lin-35(rr33) *mutant background, *hpl-1*(RNAi), *hpl-2*(RNAi) as well as *met-2*(RNAi) increased the number of multinucleate intestinal cells 2-, 2.5- and 3-fold, respectively, compared to *lin-35(rr33) *controls (Figure 3D). Another closely related methyltransferase, C47F5.1 had no apparent effect on intestinal cell development; cell division or other. This suggests that *met-2 *is likely to be the orthologue of Suv39h and it cooperates with *lin-35*/Rb and *hpl-1*/*hpl-2 *to affect changes in gene expression. These results argue that the LIN-35 repressor complex, which contains the same synMuv B genes required for proper vulva specification, also acts in the intestinal cell to promote this cell cycle transition through chromatin modification.

More recently, *lin-35 *as well as other synMuv B genes have been found to regulate RNAi sensitivity. The *E*nhancer of *R*NA*i* (*eri*) genes regulate the activity of another less well understood process that includes endoRNAi. Exactly how these genes and *lin-35*/Rb may interact mechanistically to regulate the RNAi response is still unclear [71,72]. It has recently been proposed that *dcr-1*, the only *C. elegans *dicer-like RNAse III, and other downstream components of the RNAi machinery might be rate limiting. By mutating any one of the *eri *genes the limiting pool of active *dcr-1*, as well as all the downstream components associated with this pathway, are reallocated to the active RNAi pathways, therefore making them more efficient [61,73].

Evidence suggests that the *lin-35 *repressor complex is also involved in a pathway that depends on the availability of RNAi components, but is unlikely to be involved in any functional aspect of the *eri *class of genes. First, double mutant combinations of *lin-35 *and any of the *eri *genes tested were shown to be more sensitive to RNAi than any single mutant, suggesting that they act cooperatively and potentially in parallel pathways [72]. Curiously, *lin-35; eri-3 or lin-35; rrf-3 *have a near wild-type complement of intestinal nuclei compared to *lin-35 *mutants alone, suggesting that genes in the Eri pathway can suppress the defects in the number of intestinal nuclei of the *lin-35 *mutant (35.2 ± 3.4 and 33.8 ± 2.5 respectively compared to 50.3 ± 4.7 for *lin-35(rr33) *mutant alone). We suggest that in an Eri background where the endoRNAi pathway has been disabled, RNAi limiting factors are reallocated to the synMuv B/*lin-35 *repressor pathway, therefore making it more efficient. We propose that the same process may be true to account for the enhancement of RNAi by mutation in the synMuv B components as it permits the reallocation of limiting levels of RNAi components to function in the exoRNAi pathway, therefore making it more efficient. Our results show that *dcr-1*(RNAi) enhances the *lin-35(rr33) *mutant phenotype and that by genetically increasing the availability of RNAi components to function in the synMuv B pathway we rescue the *lin-35 *mutant phenotype in the gut. We therefore propose that the synMuv B pathway requires *dcr-1 *activity, in addition to other downstream RNAi components, to properly repress gene expression, potentially through a *met-2*-directed chromatin modification.

### A model for developmentally-regulated cell cycle transitions in the intestine

**Figure 6:**
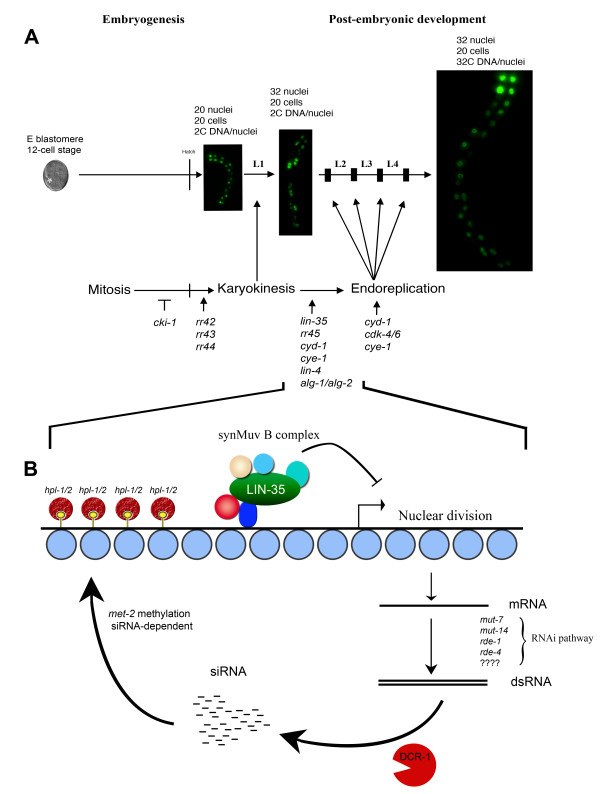
Genetic regulation of cell cycle progression in the intestinal lineage of *C. elegans*. (A) All the animals depicted express an intestinal-specific GFP marker (*elt-2::GFP)*, which is expressed from embryogenesis to the adult stage. The E blastomere is marked with a white asterisk as the transgene is not expressed at this early stage. The different types of intestinal cell cycle are marked under the schematic life cycle and the arrows indicate the developmental stage during which they occur. Black rectangles represent each molt. Anterior is up in all the figures and all the images are shown at the same magnification to represent the growth of the animal. (B) Proposed model for the *lin-35*/synMuv B complex in the regulation of the cell cycle transitions typical of the intestinal cell lineage during the L1 stage. See the text for details.

Based on our results and those of others, we propose the following model to describe how the intestinal cell divisions are controlled during *C. elegans *development (Figure 6A). From the specification of the E blastomere early during development at the 12-cell stage up to the end of embryogenesis, this precursor intestinal cell will go through five successive rounds of mitosis to give rise to 20 intestinal cells at the hatch. This is controlled by the activity of *cki-1 *and perhaps other general cell cycle regulators [29,30].

Subsequently, the posterior intestinal cells will perform a single round of nuclear division (karyokinesis) at the end of the L1 stage to become binucleate. Based on the intestinal mutant phenotype of *rr42, rr43 *and *rr44 *as well as the *lin-5 *complex, this intestinal division is likely to be positively regulated by these genes [32]. Following these nuclear divisions, all the intestinal cells perform successive rounds of endoreplication at each larval molt to become polyploid by the adult stage. We and others have shown that the transition from karyokinesis to endoreplication is controlled by the *lin-35/Rb *repressor complex, as well as a subclass of the RNAi components (This study and [35]). The defect in intestinal cell cycle transition caused by *alg-1/alg-2 *is at least partially due to its involvement in the correct processing of *lin-4*, which in turn is required for *lin-14 *downregulation at the end of the L1 stage. The gene product of *cyd-1*, *cye-1 *and the heterochronic gene *lin-4 *were also shown to cause defects in the transition from karyokinesis to endoreplication [27]. Lastly, a group of genes, like *cye-1 *and *fzr-1*, are required for endocycling in the intestinal cells in conjunction with each molt [74].

Based on our data, we suggest that the *lin-35 *repressor complex is required during the L1 molt to initiate the repression of specific genes to allow the cell cycle transition typical of this lineage. Then, through a currently uncharacterised mechanism, the production of a dsRNA molecule specific for these genes takes place, the products of which will feed into the RNAi pathway. Through the action of *dcr-1*, these dsRNA molecules will generate siRNA specific for these gene products that will feed back to the chromosome to recruit *met-2 *and promote the methylation of lysine 9 on histone 3. Once methylated, both *hpl-1 *and *hpl-2 *induce the formation of heterochromatin to stably silence these genes (Figure 6B). This mode of regulation may be analogous to the silencing process observed in yeast (*S. pombe*) and vertebrate cells where heterochromatin is established at the centromere through a dsRNA-based mechanism [64,65].

## Conclusion

We have shown that the simple switch from a proliferative mode of cell cycle to one that is more consistent with growth of the differentiated cells during development is under very strict control and that the aberrant gene expression associated with loss of either the Rb and/or the RNAi pathway can lead to defects in this switch. Although the importance of *lin-35/Rb *in regulating the cyclins implicated in this transition has been highlighted, most of the targets of the *lin-35*/Rb/RNAi pathways are yet to be elucidated [35]. Precisely how the components of the RNAi pathway and Rb coordinate these processes still remains to be characterized, but understanding how these factors contribute to the tumor suppressor function of Rb will indeed be paramount.

## Methods

### Genetics

All strains were cultured according to Brenner [75]. The following genotypes were used in this study: I: *lin-35(rr33)*(this study), *lin-35(n745); dpy-5(e61)unc-29(e1072); dpy-5(e61)unc-13(e450), dpy-5(e61)unc-13(e450); hDp41, dpy-5(e61)unc-13(e450); hDp61*. II: *lin-8(*n111) *dpy-10(e128)*. III: *mut-7(pk204)*.V: *mut-14(pk738)*. X: *rrIs01 *[*elt-2::GFP, unc-119(+)*] [30]

A genetic screen was performed using the intestinal-specific GFP (*rrIs01*) in order to identify mutants with abnormal numbers of intestinal nuclei. In summary, staged L4 animals were mutagenized using EMS at 40 μM and plated in order to produce the next generation. Then, 5 L4 F1 progeny were placed per plate and allow to produce eggs. 10 plates of gravid F2 hermaphrodites were pooled and their embryos were staged (F3). Using this approach we screened approximately 5000 haploid genomes for defects in the number of intestinal nuclei in the F3 generation.

### Lineage analysis

*lin-35(rr33); rrIs01 *adult animals were allowed to produce eggs on a seeded plate for 2–4 hours. Thereafter, newly hatched L1 larvae were placed on individual microscope slides which had a 2 mm thick NGM pad with a minimum amount of bacteria. Animals were monitored every 2–3 hours using DIC and/or epifluorescence and the slides were maintained in a humidified chamber between each examination.

### DNA quantification

DNA quantification was performed on the intestinal nuclei by fixing the adult animals with Carnoy's solution (60% EtOH, 30 % acetic acid and 10 % chloroform) overnight. The nematodes were slowly rehydrated using PBS + 0.1% Tween 20. The worms were then treated with RNAse (1 mg/ml) for 30 minutes at 37°C and stained with Propidium Iodine at 0.3 μg/ml. Z-stack series of the intestinal nuclei were taken using a Zeiss meta confocal system and the total number of pixels in each nucleus was determined using Volocity (Improvision). The ploidy of the intestinal nuclei was compared to body wall muscle nuclei.

### RNA methods

Feeding RNAi experiments were performed according to Fraser et al., 2000 [33]. In summary, L4 larvae of the corresponding genotypes were placed on bacteria induced with 1 mM of IPTG. Animals were then transferred 24 hrs later to a second plate that contained the same induced bacteria. Intestinal nuclei were then scored 48 hrs after the initial induction by monitoring the number of nuclei expressing *elt-2::GFP*. The number of intestinal nuclei for each RNAi experiment corresponds to 3 independent trials. *cki-1*(RNAi) was performed as in [30].

For semi-quantitative RT-PCR (qRT-PCR) experiments, target-specific primers for each of the corresponding genes were designed such that the upstream primer spanned an exon-intron boundary in order to discriminate between the cDNA and the genomic DNA. Each primer pair was designed to amplify regions of approximately 200 bp at the 5' end of the gene. The targets that were examined correspond to ZK507.6 (cyclin A) [rr788–rr789], ZC168.4 (cyclin B1) [rr791–rr792], Y43E12A.1 (cyclin B2.1) [rr793–rr794], H31G24.4 (cyclin B2.2) [rr795–rr796], T06E6.2 (cyclin B3) [rr797–rr798], Y38F1A.5 (cyclin D) [rr785–rr787], C37A2.4 and (cyclin E) [rr782–rr783]. The corresponding primer names are shown in the square brackets. The levels were normalized to an actin control (*act-1*, T04C12.6) [rr799–rr801], the expression of which does not change in the various mutant backgrounds.

Total RNA was isolated from L1 stage larvae of each genotype using Trizol (Invitrogen). The RNA was then run over a polyT column (Qiagen) and subsequent RT-PCR was performed on the eluate using Expand RT (Roche) (0.5–1 μg of mRNA) and a polyT primer (Invitrogen). Each PCR reaction was performed in six replicates in 384-well PCR plates using Hot Start Taq polymerase (Qiagen) and SYBR green, which was used as the reporter dye and ROX as the reference dye. The experiment was repeated 4 independent times with 4 different RT reactions. The PCR reactions were performed on an Abiprims 7900HT real-time PCR machine and the data were analyzed using SDS 2.1 software provided with the machine. Primer sequences will be provided upon request.

### Microscopy and image processing

Images were captured using an ORCA ER camera (Hamamatsu) mounted on a Leica DMR compound microscope and processed using OpenLab software (Improvision), following which the images were then transferred to Photoshop CS for assembly.

## Authors' contributions

JO and RR designed the experiments. JO carried out the experiments and drafted the manuscript. RR edited and corrected the manuscript. All authors read and approved the manuscript.

## References

[B1] Blagosklonny MV, Pardee AB (2002). The restriction point of the cell cycle. Cell Cycle.

[B2] Lee WH, Bookstein R, Hong F, Young LJ, Shew JY, Lee EY (1987). Human retinoblastoma susceptibility gene: cloning, identification, and sequence. Science.

[B3] Giacinti C, Giordano A (2006). RB and cell cycle progression. Oncogene.

[B4] Ach RA, Durfee T, Miller AB, Taranto P, Hanley-Bowdoin L, Zambryski PC, Gruissem W (1997). RRB1 and RRB2 encode maize retinoblastoma-related proteins that interact with a plant D-type cyclin and geminivirus replication protein. Mol Cell Biol.

[B5] Du W, Vidal M, Xie JE, Dyson N (1996). RBF, a novel RB-related gene that regulates E2F activity and interacts with cyclin E in Drosophila. Genes Dev.

[B6] Lu X, Horvitz HR (1998). lin-35 and lin-53, two genes that antagonize a C. elegans Ras pathway, encode proteins similar to Rb and its binding protein RbAp48. Cell.

[B7] Du W, Pogoriler J (2006). Retinoblastoma family genes. Oncogene.

[B8] Khidr L, Chen PL (2006). RB, the conductor that orchestrates life, death and differentiation. Oncogene.

[B9] Korenjak M, Brehm A (2005). E2F-Rb complexes regulating transcription of genes important for differentiation and development. Curr Opin Genet Dev.

[B10] Clarke AR, Maandag ER, van Roon M, van der Lugt NM, van der Valk M, Hooper ML, Berns A, te Riele H (1992). Requirement for a functional Rb-1 gene in murine development. Nature.

[B11] Jacks T, Fazeli A, Schmitt EM, Bronson RT, Goodell MA, Weinberg RA (1992). Effects of an Rb mutation in the mouse. Nature.

[B12] Lee EY, Chang CY, Hu N, Wang YC, Lai CC, Herrup K, Lee WH, Bradley A (1992). Mice deficient for Rb are nonviable and show defects in neurogenesis and haematopoiesis. Nature.

[B13] Dimova DK, Stevaux O, Frolov MV, Dyson NJ (2003). Cell cycle-dependent and cell cycle-independent control of transcription by the Drosophila E2F/RB pathway. Genes Dev.

[B14] Fay DS, Large E, Han M, Darland M (2003). lin-35/Rb and ubc-18, an E2 ubiquitin-conjugating enzyme, function redundantly to control pharyngeal morphogenesis in C. elegans. Development.

[B15] Fay DS, Qiu X, Large E, Smith CP, Mango S, Johanson BL (2004). The coordinate regulation of pharyngeal development in C. elegans by lin-35/Rb, pha-1, and ubc-18. Dev Biol.

[B16] Cui M, Chen J, Myers TR, Hwang BJ, Sternberg PW, Greenwald I, Han M (2006). SynMuv genes redundantly inhibit lin-3/EGF expression to prevent inappropriate vulval induction in C. elegans. Dev Cell.

[B17] Myers TR, Greenwald I (2005). lin-35 Rb acts in the major hypodermis to oppose ras-mediated vulval induction in C. elegans. Dev Cell.

[B18] Lipsick JS (2004). synMuv verite--Myb comes into focus. Genes Dev.

[B19] Fay DS, Han M (2000). The synthetic multivulval genes of C. elegans: functional redundancy, Ras-antagonism, and cell fate determination. Genesis.

[B20] Ceol CJ, Horvitz HR (2001). dpl-1 DP and efl-1 E2F act with lin-35 Rb to antagonize Ras signaling in C. elegans vulval development. Mol Cell.

[B21] Frolov MV, Dyson NJ (2004). Molecular mechanisms of E2F-dependent activation and pRB-mediated repression. J Cell Sci.

[B22] Lewis PW, Beall EL, Fleischer TC, Georlette D, Link AJ, Botchan MR (2004). Identification of a Drosophila Myb-E2F2/RBF transcriptional repressor complex. Genes Dev.

[B23] Korenjak M, Taylor-Harding B, Binne UK, Satterlee JS, Stevaux O, Aasland R, White-Cooper H, Dyson N, Brehm A (2004). Native E2F/RBF complexes contain Myb-interacting proteins and repress transcription of developmentally controlled E2F target genes. Cell.

[B24] Vandel L, Nicolas E, Vaute O, Ferreira R, Ait-Si-Ali S, Trouche D (2001). Transcriptional repression by the retinoblastoma protein through the recruitment of a histone methyltransferase. Mol Cell Biol.

[B25] Maison C, Almouzni G (2004). HP1 and the dynamics of heterochromatin maintenance. Nat Rev Mol Cell Biol.

[B26] Li Y, Kirschmann DA, Wallrath LL (2002). Does heterochromatin protein 1 always follow code?. Proc Natl Acad Sci U S A.

[B27] Boxem M, van den Heuvel S (2001). lin-35 Rb and cki-1 Cip/Kip cooperate in developmental regulation of G1 progression in C. elegans. Development.

[B28] Boxem M, van den Heuvel S (2002). C. elegans class B synthetic multivulva genes act in G(1) regulation. Curr Biol.

[B29] Hong Y, Roy R, Ambros V (1998). Developmental regulation of a cyclin-dependent kinase inhibitor controls postembryonic cell cycle progression in Caenorhabditis elegans. Development.

[B30] Kostic I, Roy R (2002). Organ-specific cell division abnormalities caused by mutation in a general cell cycle regulator in C. elegans. Development.

[B31] Tong AH, Evangelista M, Parsons AB, Xu H, Bader GD, Page N, Robinson M, Raghibizadeh S, Hogue CW, Bussey H, Andrews B, Tyers M, Boone C (2001). Systematic genetic analysis with ordered arrays of yeast deletion mutants. Science.

[B32] Srinivasan DG, Fisk RM, Xu H, van den Heuvel S (2003). A complex of LIN-5 and GPR proteins regulates G protein signaling and spindle function in C elegans. Genes Dev.

[B33] Fraser AG, Kamath RS, Zipperlen P, Martinez-Campos M, Sohrmann M, Ahringer J (2000). Functional genomic analysis of C. elegans chromosome I by systematic RNA interference. Nature.

[B34] Davison EM, Harrison MM, Walhout AJ, Vidal M, Horvitz B (2005). lin-8, which antagonizes C. elegans Ras-mediated vulval induction, encodes a novel nuclear protein that interacts with the LIN-35 Rb protein. Genetics.

[B35] Grishok A, Sharp PA (2005). Negative regulation of nuclear divisions in Caenorhabditis elegans by retinoblastoma and RNA interference-related genes. Proc Natl Acad Sci U S A.

[B36] Mango SE (2001). Stop making nonSense: the C. elegans smg genes. Trends Genet.

[B37] Zhu W, Giangrande PH, Nevins JR (2004). E2Fs link the control of G1/S and G2/M transcription. Embo J.

[B38] Coqueret O (2002). Linking cyclins to transcriptional control. Gene.

[B39] Harbour JW, Dean DC (2000). The Rb/E2F pathway: expanding roles and emerging paradigms. Genes Dev.

[B40] Stevaux O, Dyson NJ (2002). A revised picture of the E2F transcriptional network and RB function. Curr Opin Cell Biol.

[B41] Lundberg AS, Weinberg RA (1998). Functional inactivation of the retinoblastoma protein requires sequential modification by at least two distinct cyclin-cdk complexes. Mol Cell Biol.

[B42] Harbour JW, Luo RX, Dei Santi A, Postigo AA, Dean DC (1999). Cdk phosphorylation triggers sequential intramolecular interactions that progressively block Rb functions as cells move through G1. Cell.

[B43] Fitzpatrick CA, Sharkov NV, Ramsay G, Katzen AL (2002). Drosophila myb exerts opposing effects on S phase, promoting proliferation and suppressing endoreduplication. Development.

[B44] Zhang HS, Gavin M, Dahiya A, Postigo AA, Ma D, Luo RX, Harbour JW, Dean DC (2000). Exit from G1 and S phase of the cell cycle is regulated by repressor complexes containing HDAC-Rb-hSWI/SNF and Rb-hSWI/SNF. Cell.

[B45] Follette PJ, Duronio RJ, O'Farrell PH (1998). Fluctuations in cyclin E levels are required for multiple rounds of endocycle S phase in Drosophila. Curr Biol.

[B46] Edgar BA, Orr-Weaver TL (2001). Endoreplication cell cycles: more for less. Cell.

[B47] Kohn MJ, Bronson RT, Harlow E, Dyson NJ, Yamasaki L (2003). Dp1 is required for extra-embryonic development. Development.

[B48] del Pozo JC, Diaz-Trivino S, Cisneros N, Gutierrez C (2006). The balance between cell division and endoreplication depends on E2FC-DPB, transcription factors regulated by the ubiquitin-SCFSKP2A pathway in Arabidopsis. Plant Cell.

[B49] Wu L, de Bruin A, Saavedra HI, Starovic M, Trimboli A, Yang Y, Opavska J, Wilson P, Thompson JC, Ostrowski MC, Rosol TJ, Woollett LA, Weinstein M, Cross JC, Robinson ML, Leone G (2003). Extra-embryonic function of Rb is essential for embryonic development and viability. Nature.

[B50] Weng L, Zhu C, Xu J, Du W (2003). Critical role of active repression by E2F and Rb proteins in endoreplication during Drosophila development. Embo J.

[B51] Ambros V (2000). Control of developmental timing in Caenorhabditis elegans. Curr Opin Genet Dev.

[B52] Ambros V (2003). MicroRNA pathways in flies and worms: growth, death, fat, stress, and timing. Cell.

[B53] Ambros V, Moss EG (1994). Heterochronic genes and the temporal control of C. elegans development. Trends Genet.

[B54] Slack F, Ruvkun G (1997). Temporal pattern formation by heterochronic genes. Annu Rev Genet.

[B55] Slack F, Ruvkun G (1998). Heterochronic genes in development and evolution. Biol Bull.

[B56] Hong Y, Lee RC, Ambros V (2000). Structure and function analysis of LIN-14, a temporal regulator of postembryonic developmental events in Caenorhabditis elegans. Mol Cell Biol.

[B57] Olsen PH, Ambros V (1999). The lin-4 regulatory RNA controls developmental timing in Caenorhabditis elegans by blocking LIN-14 protein synthesis after the initiation of translation. Dev Biol.

[B58] Feinbaum R, Ambros V (1999). The timing of lin-4 RNA accumulation controls the timing of postembryonic developmental events in Caenorhabditis elegans. Dev Biol.

[B59] Wightman B, Ha I, Ruvkun G (1993). Posttranscriptional regulation of the heterochronic gene lin-14 by lin-4 mediates temporal pattern formation in C. elegans. Cell.

[B60] Grishok A, Pasquinelli AE, Conte D, Li N, Parrish S, Ha I, Baillie DL, Fire A, Ruvkun G, Mello CC (2001). Genes and mechanisms related to RNA interference regulate expression of the small temporal RNAs that control C. elegans developmental timing. Cell.

[B61] Duchaine TF, Wohlschlegel JA, Kennedy S, Bei Y, Conte D, Pang K, Brownell DR, Harding S, Mitani S, Ruvkun G, Yates JR, Mello CC (2006). Functional proteomics reveals the biochemical niche of C. elegans DCR-1 in multiple small-RNA-mediated pathways. Cell.

[B62] Ketting RF, Haverkamp TH, van Luenen HG, Plasterk RH (1999). Mut-7 of C. elegans, required for transposon silencing and RNA interference, is a homolog of Werner syndrome helicase and RNaseD. Cell.

[B63] Grishok A, Sinskey JL, Sharp PA (2005). Transcriptional silencing of a transgene by RNAi in the soma of C. elegans. Genes Dev.

[B64] Ekwall K (2004). The roles of histone modifications and small RNA in centromere function. Chromosome Res.

[B65] Kato H, Goto DB, Martienssen RA, Urano T, Furukawa K, Murakami Y (2005). RNA polymerase II is required for RNAi-dependent heterochromatin assembly. Science.

[B66] Volpe TA, Kidner C, Hall IM, Teng G, Grewal SI, Martienssen RA (2002). Regulation of heterochromatic silencing and histone H3 lysine-9 methylation by RNAi. Science.

[B67] Lippman Z, Martienssen R (2004). The role of RNA interference in heterochromatic silencing. Nature.

[B68] Schotta G, Ebert A, Reuter G (2003). SU(VAR)3-9 is a conserved key function in heterochromatic gene silencing. Genetica.

[B69] Couteau F, Guerry F, Muller F, Palladino F (2002). A heterochromatin protein 1 homologue in Caenorhabditis elegans acts in germline and vulval development. EMBO Rep.

[B70] Poulin G, Dong Y, Fraser AG, Hopper NA, Ahringer J (2005). Chromatin regulation and sumoylation in the inhibition of Ras-induced vulval development in Caenorhabditis elegans. Embo J.

[B71] Lehner B, Calixto A, Crombie C, Tischler J, Fortunato A, Chalfie M, Fraser AG (2006). Loss of LIN-35, the Caenorhabditis elegans ortholog of the tumor suppressor p105Rb, results in enhanced RNA interference. Genome Biol.

[B72] Wang D, Kennedy S, Conte D, Kim JK, Gabel HW, Kamath RS, Mello CC, Ruvkun G (2005). Somatic misexpression of germline P granules and enhanced RNA interference in retinoblastoma pathway mutants. Nature.

[B73] Lee RC, Hammell CM, Ambros V (2006). Interacting endogenous and exogenous RNAi pathways in Caenorhabditis elegans. Rna.

[B74] Fay DS, Keenan S, Han M (2002). fzr-1 and lin-35/Rb function redundantly to control cell proliferation in C. elegans as revealed by a nonbiased synthetic screen. Genes Dev.

[B75] Brenner S (1974). The genetics of Caenorhabditis elegans. Genetics.

